# Building research and development on poverty-related diseases

**DOI:** 10.2471/BLT.15.167072

**Published:** 2016-02-01

**Authors:** John C Reeder, Winnie Mpanju-Shumbusho

**Affiliations:** a Special Programme for Research and Training in Tropical Diseases, avenue Appia 20, 1211 Geneva 27, Switzerland.; b HIV/AIDS, Tuberculosis, Malaria and Neglected Tropical Diseases, World Health Organization, Geneva, Switzerland.

The 2015 Nobel Prize for Medicine marks an important milestone for research on infectious diseases of poverty. The award was shared by researchers identifying novel therapies for infections caused by roundworm parasites and malaria. This is important not only because of the excellent science that was conducted to achieve these discoveries, but because the prizes were given for diseases affecting the poorest and most vulnerable members of society.

The Nobel Prize Committee said: “These two discoveries have provided humankind with powerful new means to combat these debilitating diseases that affect hundreds of millions of people annually. The consequences in terms of improved human health and reduced suffering are immeasurable.”

Despite these gains, huge gaps remain. As we celebrate the Nobel Prizes, it’s also time to reflect on the lessons learnt and to highlight what is still needed for a more equitable approach to research and development.

Chinese scientist Youyou Tu was recognized for the rediscovery of artemisinin, which led to the artemisinin-based combination treatments for malaria that have saved millions of lives. Satoshi Ōmura from Japan and William Campbell from the United States of America (USA) discovered avermectin, which led to the development of an effective treatment against river blindness and lymphatic filariasis, diseases that cause immense suffering and severe disfigurement.

In both examples, these discoveries of the 1970s did not lead directly to public health impact. That required the further commitment of the United Nations, and the development of organizations dedicated to building research capacity for neglected diseases, such as the Special Programme for Research and Training in Tropical Diseases. In addition, partnerships were formed between international research organizations and pharmaceutical companies. Non-profit product development partnerships such as the Medicines for Malaria Venture and the Drugs for Neglected Diseases initiative were later formed, to focus on the development of drugs and diagnostics specifically in low-income countries.

For Youyou Tu, linkages spanned China, Africa, Europe and the USA. There was critical support from international pharmaceutical companies to develop the new treatment to international standards and to run multi-country clinical drug trials. Scientists from low- and middle-income countries across Africa were trained and a critical generation of research capacity was begun.

Once the treatments were found to be safe and effective, research on packaging was needed to ensure that illiterate people could understand the dosages. New policies, both by the World Health Organization (WHO) and the national health ministries, were required. Research identified new community-based approaches that ensured treatment was available to those who needed them, where they needed them.

Today, artemisinin compounds are the mainstay of malaria treatment. Since 2000, more than 1 billion artemisinin-based treatment courses have been administered to over 600 million people,^1^ which has contributed to the impressive 54% fall in malaria cases in the African region.^1^

Treatment for river blindness and lymphatic filariasis benefited from a similar public–private research collaboration. Discovery of avermectin led to the development of the related drug ivermectin. Clinical trials were conducted in Africa by Africans. However, the key to widespread use was research on social systems, gender and economics showing how communities could lead the distribution of ivermectin.

By 2013, more than 100 million people in sub-Saharan Africa were receiving annual treatments of ivermectin for onchocerciasis,^2^ ending the era when many people in Africa were blinded or severely disfigured from onchocerciasis. A new programme to eliminate onchocerciasis has now been created.

Unfortunately, despite these successes, there are still gaps. New products to fight drug resistance and new distribution strategies are needed. On a positive note, nearly 500 product candidates are in the pipeline for neglected diseases, including treatments, diagnostics and vaccines.^3^ About three-quarters of these have been developed through public–private product development partnerships.

The challenge now is to ensure that scientific and political momentum is not only sustained but expanded and, importantly, coordinated and financed.^4^ At a recent G7 Summit, science ministers committed to extending research on infectious diseases of poverty.^5^ New mechanisms, such as the WHO Global Observatory on Research and Development and the proposed Global Research and Development Fund, will be needed to define and fund public health priorities.

Today, we need to sustain the research and development system that has been built from these prize-winning discoveries. Achieving impact required a full spectrum of research from discovery to product development to implementation. Innovative new partnerships, industry support and political will were necessary to fund the work (including the resulting treatments). Now, a globally coordinated response is needed to ensure that more great discoveries and innovation reach the people who need them, no matter where they live.
