# Corrigendum

**DOI:** 10.2471/BLT.16.110416

**Published:** 2016-04-01

In Volume 94, Issue 2, February 2016, page 78, the bottom line should read: “Correspondence to John C Reeder (email: reederj@who.int).”

